# Control of *Xenopus* Tadpole Locomotion via Selective Expression of Ih in Excitatory Interneurons

**DOI:** 10.1016/j.cub.2018.10.048

**Published:** 2018-12-17

**Authors:** Laurence D. Picton, Keith T. Sillar, Hong-Yan Zhang

**Affiliations:** 1 School of Psychology and Neuroscience, University of St Andrews, St Andrews KY16 9JP, UK; 2 Centre for Discovery Brain Sciences, Edinburgh Medical School, University of Edinburgh, Edinburgh EH16 4SB, UK

**Keywords:** Ih, HCN channels, afterhyperpolarization, Na/K pump, *Xenopus*, central pattern generator

## Abstract

Locomotion relies on the coordinated activity of rhythmic neurons in the hindbrain and spinal cord and depends critically on the intrinsic properties of excitatory interneurons. Therefore, understanding how ion channels sculpt the properties of these interneurons, and the consequences for circuit function and behavior, is an important task. The hyperpolarization-activated cation current, Ih, is known to play important roles in shaping neuronal properties and for rhythm generation in many neuronal networks. We show in stage 42 *Xenopus laevis* frog tadpoles that Ih is strongly expressed only in excitatory descending interneurons (dINs), an important ipsilaterally projecting population that drives swimming activity. The voltage-dependent HCN channel blocker ZD7288 completely abolished a prominent depolarizing sag potential in response to hyperpolarization, the hallmark of Ih, and hyperpolarized dINs. ZD7288 also affected dIN post-inhibitory rebound firing, upon which locomotor rhythm generation relies, and disrupted locomotor output. Block of Ih also unmasked an activity-dependent ultraslow afterhyperpolarization (usAHP) in dINs following swimming, mediated by a dynamic Na/K pump current. This usAHP, unmasked in dINs by ZD7288, resulted in suprathreshold stimuli failing to evoke swimming at short inter-swim intervals, indicating an important role for Ih in maintaining swim generation capacity and in setting the post-swim refractory period of the network. Collectively, our data suggest that the selective expression of Ih in dINs determines specific dIN properties that are important for rhythm generation and counteracts an activity-dependent usAHP to ensure that dINs can maintain coordinated swimming over a wide range of inter-swim intervals.

## Highlights

• Ih is strongly expressed in *Xenopus* locomotor-rhythm-generating dIN interneurons
• Ih is active at rest in dINs, contributing to their distinct electrical properties
• dINs normally lack a Na pump-dependent ultra-slow afterhyperpolarization (usAHP)
• Ih counterbalances dIN usAHPs to preserve tadpole rhythm generating capacity

## Teaser

Picton et al. reveal that a hyperpolarization-activated current, Ih, selectively affects the electrical properties of locomotor rhythm generating interneurons in the *Xenopus* tadpole spinal motor circuit. By counterbalancing a dynamic hyperpolarizing Na pump current, Ih preserves rhythm generating capacity at short inter-swim intervals.

## Introduction

The constituent neurons of neural networks, such as those controlling rhythmic locomotor behaviors, are connected by complex synaptic interactions and express a wide range of ion channels that regulate their intrinsic electrical properties. These networks are influenced by an assortment of neuromodulators that target and modify specific ionic conductances and synaptic strengths. One important ionic current involved in network rhythmicity, which is also subject to neuromodulation, is “Ih”; the hyperpolarization-activated cation current. Ih is mediated by cyclic nucleotide-gated (HCN) channels, of which there are four mammalian subunit isoforms (HCN1–4) that become activated by membrane potential hyperpolarization at levels more negative than −40 to −50 mV [1]. Ih currents were discovered in the rabbit heart sinoatrial node [2] but are now known to be present in many cell types and often contribute to the rhythmogenic properties of neuronal networks [3, 4, 5]. At its simplest, the presence of a resting Ih current can stabilize the membrane potential and decrease neuronal input resistance [4, 5, 6]. Often, however, Ih also plays a more complex role, being activated by precisely timed hyperpolarizing inputs, whose resulting activation generates a dynamic depolarization to provide an escape from inhibition that is critical to the rhythmicity of many pacemaker neuron types [4, 5, 6]. Furthermore, through its effect on intrinsic properties, Ih currents can shorten the duration of incoming post-synaptic potentials (PSPs), which in turn facilitates precise integration [4, 5, 6].

The contribution of Ih to rhythm generation has been studied extensively in invertebrate networks. For example, Ih contributes to the rhythmic firing of leech heart interneurons (HNs) by facilitating rebound spiking [7], and it plays a similar role in pyloric neurons of the crustacean stomatogastric ganglion (STG), where it is also targeted by various neuromodulators [8, 9]. In the marine gastropod, *Clione limacina*, Ih currents trigger post-inhibitory rebound in interneurons controlling their wing-like parapodia during swimming [10]. Ih is also consistently found in various rhythmically active networks in vertebrates [11, 12, 13], where there is evidence for a contribution of Ih toward maintaining rhythmic locomotor network activity [14]. Furthermore, Ih has been documented in neurons in spinal motor networks in turtle [15] and rat [16, 17, 18].

The swimming rhythm in hatchling *Xenopus* tadpoles (stage 37-38) relies on a post-inhibitory rebound mechanism in excitatory descending interneurons (dINs) following phasic mid-cycle inhibition from glycinergic commissural interneurons (cINs) [19]. Neither the full range of currents dictating the distinct intrinsic properties of dINs nor the specific mechanism of post-inhibitory rebound in dINs have yet been fully described, although the de-inactivation of fast, voltage-gated sodium channels and riluzole-sensitive persistent sodium currents is thought to be partly involved in the rebound mechanism [20, 21]. Other ionic conductances, especially Ih, could also contribute to the intrinsic properties of dINs, including rebound firing, and therefore locomotor rhythm generation. We have also documented in previous studies that dINs are the only neuron class in the *Xenopus* locomotor central pattern generator (CPG) not to display an activity-dependent Na/K pump current that generates an ultraslow afterhyperpolarization (usAHP) following the end of each swim episode [22, 23]. Interestingly, Ih is known to be a common interacting partner with Na/K pump currents in diverse cell types [24, 25, 26, 27, 28, 29, 30].

Here, we show that Ih is specifically expressed and active at rest in the rhythm-generating dINs that control swimming in *Xenopus* tadpoles. When Ih is blocked by ZD7288, dINs hyperpolarize by ∼10 mV and their input resistance increases. The presence of Ih in dINs is important for maintaining stable locomotor rhythm generation; ZD7288 results in shorter swim episodes displaying more variable cycle frequencies, motor bursts, and left-right alternation. ZD7288 also diminishes dIN rebound firing from rest and prevents single dIN action-potential-induced swimming. Surprisingly, Ih masks a Na/K-pump-dependent usAHP that could otherwise silence dINs at the end of a swim episode and impair the initiation of subsequent swimming, as evidenced by ZD7288 causing swim failure at longer than normal inter-swim intervals. Thus, Ih plays a critical role in swimming in *Xenopus* tadpoles by facilitating rhythmic firing in dINs, holding dINs more depolarized to maintain excitability, and controlling short-term motor memory.

## Results

### Larval Excitatory dINs Display Unique Properties

The rhythmic output from CPG networks is usually driven by subpopulations of excitatory interneurons, which often have properties that distinguish them from other network neurons [31]. In *Xenopus* tadpoles, phasic excitation within the swim CPG network derives from a discrete population of ipsilaterally projecting dINs. Previous studies on dINs have focused on embryonic stage 37–38 [32]. Embryonic dINs display pacemaker-like properties and differ from other CPG neuron types in their firing pattern during swimming, broad action potential shape, and relatively depolarized resting membrane potential (RMP) [32, 33]. The dINs are also the only neuron in the *Xenopus* swim network not to display an activity-dependent and Na/K-pump-mediated usAHP [22, 23]. To test whether these unique characteristics of dINs continue through development and to further reveal the role of dINs in locomotor rhythm generation, we examined dINs at the more mature larval stage 42 [34].

**Figure 1:**
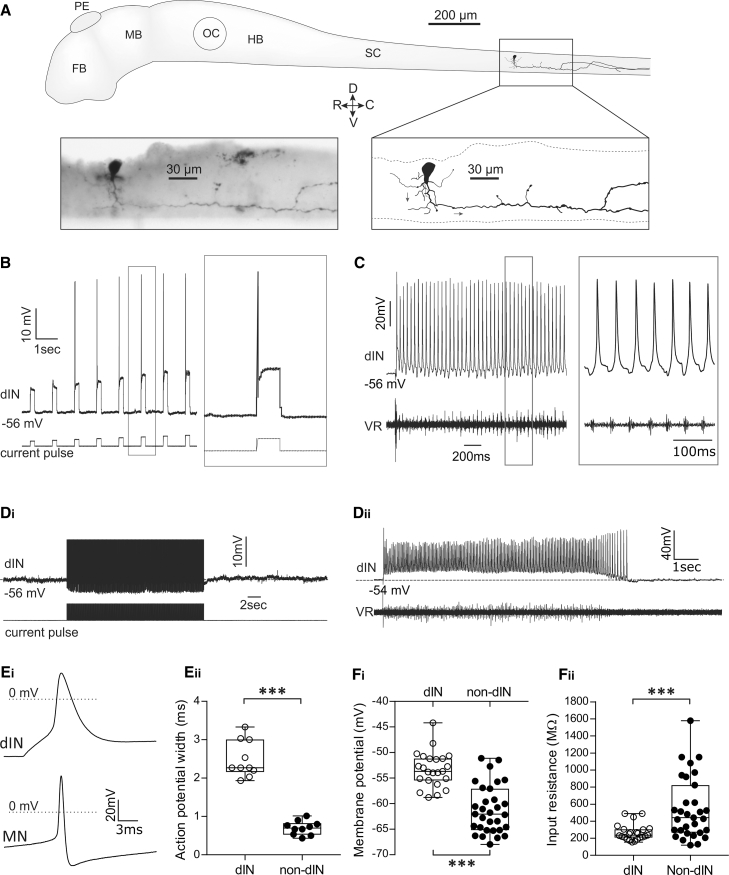
Properties of dINs in Stage 42 Larval *Xenopus* Tadpoles (A) The anatomy of a dIN. The axon courses ventrally and then caudally. Arrows on the image indicate the direction of the axon. FB, forebrain; HB, hindbrain; MB, midbrain; OC, otic capsule; PE, pineal eye; SC, spinal cord. (B) Responses of a dIN to depolarizing current pulses of increasing amplitude. Unlike non-dINs, suprathreshold pulses at all amplitudes generate only a single spike. (C) During swimming, dINs also only fire a single spike per swim cycle. VR, ventral root. (D) Unlike all other neuron types in the *Xenopus* spinal motor circuit, dINs never display a usAHP in response to a protocol inducing repetitive spiking (Di) or after swimming (Dii). Note that action potentials in (Di) have been truncated. (E) The action potential shape of larval dINs is different from non-dINs. Examples of a dIN and motoneuron (MN) action potential in response to a suprathreshold depolarizing pulse (Ei) are shown. Pooled action potential width of dINs and non-dINs measured at 0 mV (Eii; n = 10; ^∗∗∗^p < 0.001; median with 50% interquartile range (IQR) displayed as box-and-whisker plots). (F) The intrinsic properties of dINs (n = 28) differ from non-dINs (n = 31) and display a significantly more depolarized RMP (Fi) and significantly lower input resistance (Fii; median with 50% IQR displayed as box-and-whisker plots). ^∗∗∗^p < 0.001. See also Figure S1.

In contrast to all other classes of swim CPG neuron, whose properties change dramatically during early larval life [35], we find that dINs in stage 42 tadpoles are essentially indistinguishable from their late embryonic stage 37-38 counterparts. Anatomically, larval dINs, as in the embryo, still possess a long, descending axon ipsilateral to the soma (Figures 1A and S1), and physiologically, they continue to fire only a single action potential in response to suprathreshold depolarizing pulses (Figure 1B) and in each swim cycle (Figure 1C; n = 39). As at stage 37-38 [22], stage 42 dINs apparently lack a usAHP following either suprathreshold depolarizing pulse trains (Figure 1Di) or swimming episodes (Figure 1Dii). Furthermore, larval dIN action potentials remain much broader than those of non-dINs (Figure 1Ei). The width of action potentials evoked by injected currents as measured at 0 mV was 2.5 ± 0.5 ms for dINs (n = 10) compared with 0.7 ± 0.2 ms for non-dINs (n = 10; p < 0.001; Figure 1Eii). Consistent with a previous publication on late embryonic tadpoles [33], larval dINs are also significantly more depolarized (dIN = −52.6 ± 4.2 mV, n = 28 versus non-dIN = −61.2 ± 4.7 mV, n = 31; p < 0.001; Figure 1Fi) and have lower input resistances (269.6 ± 91.3 MΩ; n = 28), compared to a sample of non-dINs (545.2 ± 362.3 MΩ; n = 31; p < 0.001; Figure 1Fii). These latter properties suggest that a depolarizing ionic conductance, such as Ih, might be more active at rest in dINs than in other spinal cell types.

### Heterogeneous Expression of Ih among CPG Neurons

To test for the presence of Ih indirectly, we examined whether neurons displayed a slowly activating depolarizing sag potential in response to long hyperpolarizing current pulses, the hallmark characteristic of Ih activation. We applied 3-s hyperpolarizing pulses of increasing amplitude (10 or 30 pA incremental steps) to both larval dINs and non-dINs.

**Figure 2:**
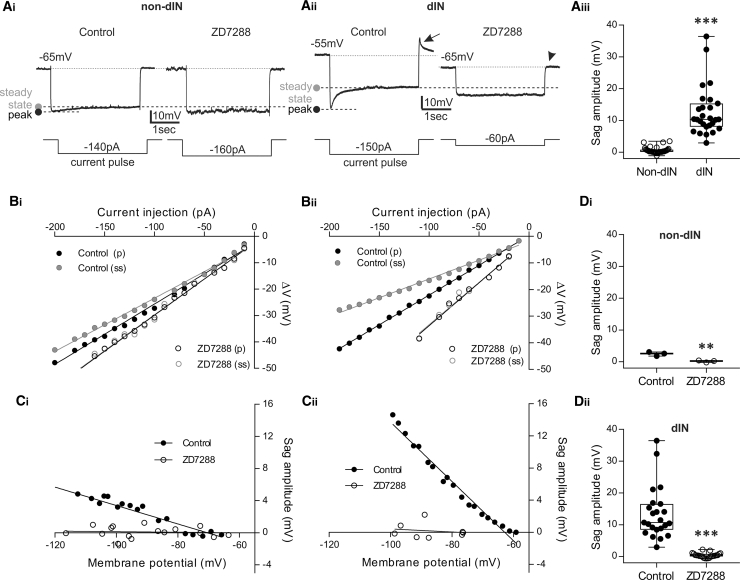
Ih Is Present in All dINs and in Some Non-dINs and Is Blocked by ZD7288 (A) An example of a sag potential in a non-dIN (Ai) with and without ZD7288 (50 μM). The peak and steady-state membrane potential responses are indicated by dashed lines. (Aii) An example response of a dIN to hyperpolarizing current pulses (3-s duration). ZD7288 (10 μM) hyperpolarized the RMP (see also Figure 3) and abolished the large sag potentials observed at hyperpolarized membrane potentials. Arrow, post-inhibitory depolarization seen in control; arrow head, the membrane potential rebound was absent in the presence of ZD7288. (Aiii) Pooled data showing a comparison of the sag amplitude between −80 and −90 mV in dINs (n = 27) and non-dINs (n = 34; ^∗∗∗^p < 0.001; median with 50% IQR displayed as box-and-whisker plots). (B) The voltage-current (V-I) relationship for a non-dIN (Bi) and a dIN (Bii) in response to hyperpolarizing current pulses (3-s duration) of increasing amplitude (10 pA incremental steps) and the effect of 10–50 μM ZD7288. For both control (closed circles) and in ZD7288 (open circles), the peak (p) and the steady-state (ss) membrane potential change are plotted against each injected current amplitude. (C) The amplitude of the sag potentials in a non-dIN (Ci) and a dIN (Cii) was plotted against the membrane potential with (open circles) or without (closed circles) ZD7288. (D) Pooled data showing that ZD7288 (10–50 μM) significantly abolished the sag potential observed around −80 mV. Data are expressed as median with 50% IQR and displayed as box-and-whisker plots with individual data points. (Di): 3 non-dINs, p = 0.0048; (Dii): 23 dINs, p < 0.001; ^∗∗^p < 0.01; ^∗∗∗^p < 0.001. See also Figure S2.

In a sample of 45 non-dINs tested, 24% of neurons showed no evidence of a slow sag potential from rest to around −120 mV (n = 11/45). For the other 76% of non-dIN neurons (n = 34/45), small sag potentials did appear in response to hyperpolarization but only at extremely hyperpolarized, non-physiological membrane potentials (mean sag appearance: −85.9 ± 10.1 mV; n = 34; e.g., Figures 2Ai, 2Bi, and 2Ci). An example of the peak and steady-state membrane potential changes upon hyperpolarizing pulses of increasing amplitude is shown in Figure 2Bi. To confirm that these sag potentials are indeed mediated by the activation of Ih current, we applied the selective Ih blocker ZD7288 at concentrations reported to specifically block HCN channels with little or no off-target effects (≤50 μM; e.g., [25, 36]). These small sag potentials were clearly and significantly blocked by 50 μM ZD7288 (Figures 2Ai, 2Bi, 2Ci, and 2Di; 2.5 ± 0.6 mV versus 0.2 ± 0.3 mV; n = 3; p = 0.0048), demonstrating that they are most likely mediated by HCN channels. This resulted in a rightward shift in the voltage-current (V-I) relationship (Figure 2Bi), but this was only apparent at very hyperpolarized membrane potentials, supporting the hypothesis that Ih is not active at rest in these non-dINs. Indeed, ZD7288 (50 μM) had no clear effect on the RMP (Figures S2A, S2B, and S2Ci; −59.6 ± 5.8 mV versus −59.1 ± 7.1 mV; n = 3; p = 0.93). Similarly, there was no clear change in resting input resistance (Figures S2B and S2Cii; 711.5 ± 765.5 MΩ versus 705.4 ± 723 MΩ; n = 3; p = 0.99).

**Figure 3:**
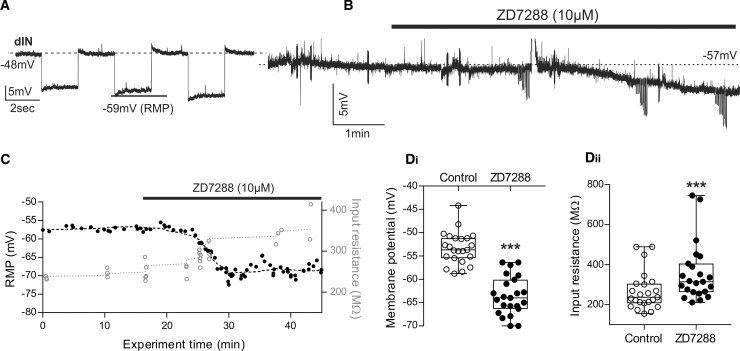
Ih Current Is Active at Rest and Contributes to the Intrinsic Properties of dINs (A) When dINs were held depolarized (∼−48 mV), hyperpolarizing pulses revealed a sag response around the original RMP. (B) Raw trace on a slow time base showing a clear membrane hyperpolarization of approximately 10 mV following the application of ZD7288 (10 μM). Blockade of Ih hyperpolarized all dINs. (C) Resting membrane potential (RMP) and input resistance plotted against experiment time for the experiment shown in (B). The hyperpolarization was accompanied by an increase in input resistance. Dashed line indicates the change of IR of the dIN in (B). (D) Pooled paired data showing that (Di) the membrane potential of dINs was significant hyperpolarized by ZD7288 (n = 23; p < 0.001; median with 50% IQR displayed as box-and-whisker plots). (Dii) Pooled paired data show a significant increase in input resistance in ZD7288 (n = 23; p < 0.001; median with 50% IQR displayed as box-and-whisker plots). ^∗∗∗^p < 0.001. See also Figure S2.

In contrast, in every dIN recording (41/41), there was a prominent sag potential in response to hyperpolarizing pulses (Figures 2Aii, 2Bii, and 2Cii). Importantly, these sag potentials consistently appeared even with only moderate hyperpolarization, close to the RMP (mean sag appearance: −60.1 ± 4.5 mV; n = 21). An example of the membrane potential changes upon increasing current pulses is shown in Figure 2Bii, and a difference between peak and steady-state potentials can be seen upon small-amplitude hyperpolarizing pulses. The dIN sag potential amplitude was 12.7 ± 7.7 mV (n = 27) at approximately −80 mV, and this was significantly larger than that of non-dINs (excluding those without a sag), which was close to 0 mV at this membrane potential (Figure 2Aiii; 0.6 ± 1 mV; n = 34; p < 0.001). Again, ZD7288 (10–50 μM) significantly reduced this sag potential to 0.4 ± 0.7 mV (Figures 2Cii and 2Dii; n = 23; p < 0.001). A small post-inhibitory depolarization observed in dINs under control conditions (Figure 2Aii, arrow) was also abolished by ZD7288 (Figure 2Aii, arrow head). In addition, ZD7288 shifted the V-I relationship to the right (Figure 2Bii), indicating that block of Ih increases the voltage response to a given current step. This in turn illustrates an increase in resting input resistance (see also Figure 3) and provides further evidence that ZD7288 blocks active Ih in these neurons.

### Block of a Resting Ih Current Affects dIN Properties

In dINs, sag potentials appeared even at moderately hyperpolarized levels, suggesting that Ih may be active at rest. Therefore, dINs were held at more depolarized membrane potentials, between −40 and −50 mV, hyperpolarizing pulses produced clear and prominent sag potentials around the RMP (mean sag appearance −52.7 ± 4.1 mV; Figure 3A; n = 11), indicating that Ih is indeed active around the RMP and could contribute to passive dIN properties.

We then tested whether ZD7288 affects the RMP and input resistance of dINs. Following the application of 10 μM ZD7288, the RMP hyperpolarized (Figure 3B; see also Figure 2Aii), and when the same data were plotted against time in Figure 3C, a simultaneous increase in input resistance is evident. On average, ZD7288 (10–50 μM) hyperpolarized the RMP from −53.3 ± 3.4 mV to −63.7 ± 4.5 mV (Figure 3Di; n = 23; p < 0.001) and increased input resistance from 267.8 ± 96 MΩ to 356.7 ± 143.4 MΩ (Figure 3Dii; n = 23; p < 0.001). These results support the hypothesis that Ih is active at rest in dINs and contributes to their more positive RMP and lower input resistance compared to other neurons in the swim network.

### Stable Swim Network Output Is Disrupted by Block of Ih

**Figure 4:**
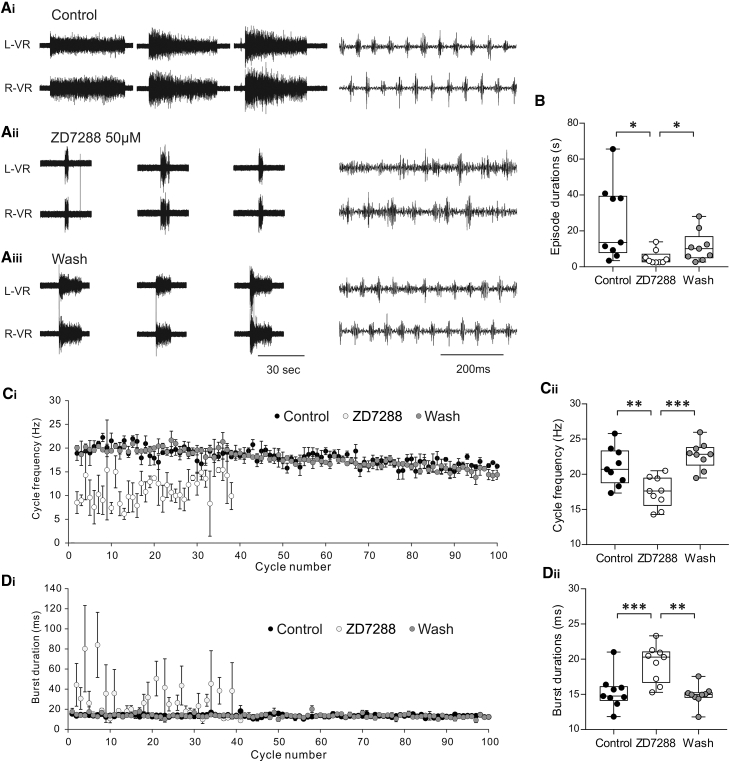
The Effects of ZD7288 on *Xenopus* Swim Network Output (A) Two simultaneously recorded raw ventral root traces on the left and right sides showing evoked swim episodes in control (Ai), in the presence of the Ih current blocker ZD7288 (50 μM; Aii), and after washout (Aiii). The right side panels show an expansion of fictive swimming activity. (B) ZD7288 (10–50 μM) significantly shortened episode duration (p = 0.026), and the effect was reversed following washout of ZD7288 (n = 9 complete experiments; p = 0.027; median with 50% IQR displayed as box-and-whisker plots). (C) Time plot showing (Ci) mean swim cycle frequency across 3 evoked episodes in control, ZD7288, and after washout. Note that the swim frequency is lower and more variable. (Cii) ZD7288 (50 μM) caused a significant decrease in cycle frequency (p = 0.0008; n = 9; median with 50% IQR displayed as box-and-whisker plots). (D) Time plot showing (Di) mean burst durations across 3 evoked episodes in control, ZD7288, and following washout. (Dii) ZD7288 (50 μM) caused a significant increase in burst duration (p = 0.0018; n = 9; median with 50% IQR displayed as box-and-whisker plots). ^∗^p < 0.05; ^∗∗^p < 0.01; ^∗∗∗^p < 0.001. See also Figure S3.

As blocking Ih currents affects the intrinsic properties of dINs, which coordinate and maintain swimming, block of Ih should in turn affect swimming activity. Therefore, we explored the effects of blocking Ih currents with ZD7288 on swim network output using extracellular ventral root recordings and report clear effects of ZD7288 on a number of swimming parameters. First, ZD7288 (50 μM) significantly shortened the duration of evoked swimming episodes (Figures 4A and 4B; 25.2 ± 21.2 s versus 5.1 ± 4 s; p = 0.03; n = 9). Second, ZD7288 (50 μM) affected the intrinsic parameters of swimming episodes. Although the coordination of the rhythm was not totally disrupted (Figures 4Aii and S3A), left-right alternation became much more variable (Figure S3B), as did the cycle frequencies and burst durations (Figures S3C and S3D), with disruption of individual bursts observed during the period of the drug application (Figures 4Aii and 4Di). Overall, ZD7288 effects were manifested as a significant decrease in swim frequency (Figure 4C; 21.1 ± 2.7 Hz versus 17.5 ± 2.2 Hz; p = 0.002; n = 9) and increase in burst durations (Figure 4D; 15.4 ± 2.5 ms versus 19.4 ± 2.7 ms; p < 0.001; n = 9). These changes were significantly reversed following washout of ZD7288 (episode duration: 11.3 ± 8.5 s, p = 0.03; frequency = 22.7 ± 1.9 Hz, p < 0.001; burst duration = 14.9 ± 1.5 Hz, p = 0.006). These results demonstrate that the block of Ih, which primarily affects dIN properties, potently influences the duration of swimming bouts and the properties of individual swimming bursts.

### Block of Ih Affects dIN Firing during Swimming

**Figure 5:**
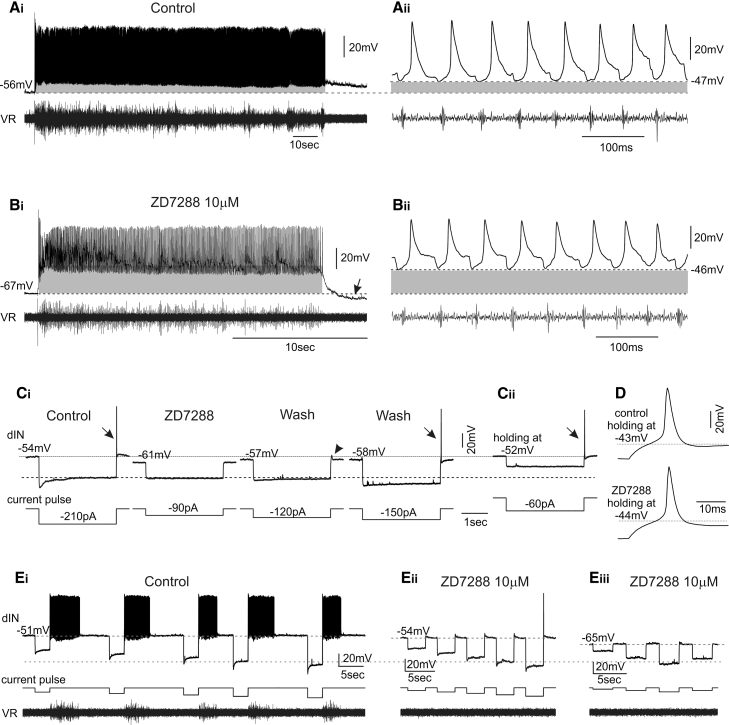
Blocking Ih Currents Alters dIN Firing during Swimming and Affects Rhythm Initiation (A) Example of a dIN firing during swimming. (B) In the presence of ZD7288, the tonic depolarization indicated by the gray area is larger and dINs fire at a more depolarized level. Note the different timescales in (Ai) and (Bi) and the shorter episode duration in the presence of ZD7288. The action potentials shown in (Bii) are smaller than those in (Aii). Arrow indicates a membrane hyperpolarization following the end of the swimming episode. (C) Membrane responses to (Ci) current pulses in control, in the presence of the Ih blocker ZD7288 (10 μM), and in wash. ZD7288 abolished both the sag currents and the PIR action potentials (arrow). Its effect could be partially reversed after a long wash. The post-inhibitory depolarization seen in control disappeared in the presence of ZD7288 and re-appeared during wash (arrow head). (Cii) In the presence of ZD7288, the PIR action potential could be induced when holding RMP to the control level by current injection. (D) The rebound action potential shapes in control condition and in ZD7288. (E) In some dINs (n = 3), (Ei) a single rebound action potential induced by a hyperpolarizing pulse initiated fictive swimming activity (note ventral root [VR] activity). (Eii) The property shown in (Ei) was abolished soon after ZD7288 was applied (approx. 10 min after drug application; n = 3), although PIR firing could still be evoked and the sag potential is only partially blocked. (Eiii) Later in ZD7288 treatment, both the PIR firing and the sag potential were totally abolished.

The disruption of locomotor output following block of Ih might be due to the contribution of Ih to dIN intrinsic properties and consequently dIN firing during swimming. In the presence of 10 μM ZD7288, the Ih current was fully blocked in the recorded dINs, but they were still able to fire rhythmically during swimming, similar to the control condition (Figures 5A and 5B; note different timescales in Figures 5Ai and 5Bi). This is most likely due to only superficial dINs, including the recorded dIN, being fully exposed to ZD7288 because of the shorter application (about 15 min) and lower concentration of ZD7288 used for these patch experiments in order to obtain an effective drug washout. However, the tonic depolarization during swimming was noticeably larger (see the gray areas in Figures 5A and 5B) and dINs fired from a significantly more depolarized level (−42.5 ± 10.1 mV versus −48.3 ± 7 mV; n = 15; p < 0.001). This in turn reduced the amplitude of action potentials (Figures 5Aii and 5Bii) and could alter the timing of post-inhibitory rebound (PIR) firing, changes likely to affect network output [37] if a sufficient number of dINs were affected simultaneously.

In response to hyperpolarizing current pulses from rest, dINs produced a post-inhibitory depolarization (Figure 2Aii, arrow), which, with large-amplitude pulses, was sufficient to induce an action potential (Figure 5C, arrow). This PIR action potential was abolished in the presence of 10 μM ZD7288 (Figure 5Ci; n = 11), similar to the blockade of post-inhibitory depolarization (Figures 2Aii and 5Ci, arrow head). Such an effect was reversible together with a re-appearance of Ih following washout of ZD7288 (Figure 5Ci). Therefore, Ih may be involved in the coordination of the swimming rhythm by supporting PIR spiking, affecting swim rhythm generation on a cycle-by-cycle basis. However, when the RMP was depolarized, the hyperpolarizing pulses were able to induce PIR action potentials again, even in the presence of ZD7288 (Figure 5Cii). This indicates that other ionic conductances are also involved in PIR firing during swimming. Although Ih affects dIN intrinsic properties, the action potential shapes evoked by current pulses were similar (Figure 5D; 1.9 ± 0.6 ms versus 1.9 ± 0.7 ms; n = 3), indicating that ZD7288 has little or no effect on Na^+^, Ca^2+^, or K^+^ channels mediating action potentials.

In some dINs, single action potentials evoked by current injection, including the PIR firing from rest, were sufficient to induce fictive swimming (n = 3); a similar phenomenon has been previously reported in the *Xenopus* embryo CPG network [38]. Figure 5Ei shows such an example, which was abolished by 10 μM ZD7288 (Figure 5Eii), even when Ih was only partially blocked. PIR action potential firing could still be induced by much larger hyperpolarizations, but no network activity could be evoked by these PIR spikes (Figure 5Eii). Later in these experiments, Ih was completely blocked and PIR could no longer be induced (Figure 5Eiii).

### A usAHP Was Unmasked in dINs by Blocking Ih

**Figure 6:**
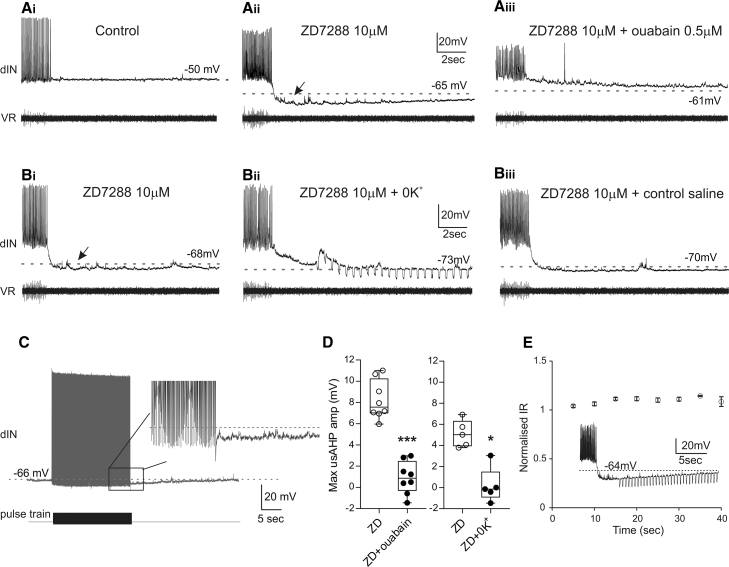
Blocking Ih Revealed a Post-swim usAHP (A) Following dIN firing in control, (Ai) the membrane potential repolarized to the baseline. (Aii) In the presence of 10 μM ZD7288, the RMP hyperpolarized (dashed line) and a long-lasting AHP appeared following the end of a swimming episode. (Aiii) The usAHP was abolished after adding 0.5 μM ouabain in the bath. (B) Another dIN displayed a usAHP in the presence of ZD7288 (Bi). Removing K^+^ ions from saline abolished the usAHP (Bii). After replacing 0K^+^ saline with control saline, the usAHP reappeared (Biii). Dotted lines in (A) and (B) indicate the resting membrane potentials. Downward deflections in (Bii) are conductance pulses. (C) A train of depolarizing pulses that mimics swimming was applied to dINs following ZD7288 treatment. A small afterhyperpolarization was observed in some dINs (8 out 12), which was never observed in control (see Figure 1Di). (D) Pooled data indicate that the maximum amplitude of usAHP was reduced significantly by 0.5 μM ouabain (left panel; n = 8; ^∗∗∗^p < 0.001) or 0K^+^ saline (right panel; n = 5; ^∗^p = 0.011). Data are expressed as median with 50% IQR and displayed as box-and-whisker plots with individual data points. (E) The input resistance (IR) during the usAHP period was tested and plotted against time. The inset shows an example of current pulses injected during usAHP period. Pooled data indicate that there was no significant change in input resistance during the usAHP (n = 3; one-way ANOVA; p = 0.26; mean ± SD).

We also observed an unexpected but important additional effect on the membrane properties of dINs, which may also partly account for the effects of Ih blockade on the swim network output. In the presence of 10 μM ZD7288, a clear long-lasting membrane hyperpolarization was seen in all dINs (Figures 5Bi, 6Aii, 6Bi, and 6D; mean amplitude = 6.5 ± 2.2 mV; n = 23) following a swimming bout. With an average duration of 37.3 ± 9 s, this AHP resembles the usAHP in non-dINs [22]. Furthermore, when a 20-s-long suprathreshold depolarizing pulse train (25 ms; 20 Hz) was applied in the presence of ZD7288 to mimic swimming activity, with each pulse evoking a dIN spike, a small, long-duration AHP was seen in the majority of dINs at the end of the spike train (Figure 6C; 8 out of 12 dINs). Neither pulse-train-induced AHPs nor the AHPs following swimming were ever observed in dINs under control conditions (Figures 1D, 5Ai, and 6Ai) [22].

We have previously shown that the usAHP in non-dINs is mediated by the recruitment of dynamic Na/K pumps, which modulate the excitability of the CPG network and act as a form of short-term motor memory [22]. To test whether this unmasked dIN usAHP is also mediated by a dynamic Na/K pump current, 0.5 μM ouabain (a specific Na/K pump blocker) was applied in the presence of ZD7288. This abolished the usAHP completely (Figures 6Aiii and 6D; 8.2 ± 1.8 mV versus 0.9 ± 1.5 mV; n = 8; p < 0.001). To confirm this result, zero K^+^ saline was applied in the presence of ZD7288, because removing K^+^ ions from the saline can arrest Na/K pump activity. Indeed, the zero K^+^ saline reversibly abolished the usAHP unmasked by ZD7288 (Figures 6Bii, 6Biii, and 6D; 5.1 ± 1.3 mV versus 0.2 ± 1.7 mV; n = 5; p = 0.011), which also excludes the involvement of K^+^ currents in the usAHP. The input resistance during the usAHP period was also tested; there should be no change in input resistance during the usAHP if it is mediated by pumps rather than ion channels. Repetitive small hyperpolarizing pulses were injected, as shown in the examples of Figures 6Bii and 6E inset. The input resistance was plotted against time in Figure 6E, and no significant input resistance change was found (n = 3; p = 0.13), indicating no net ion channel opening or closing during the period of the usAHP. This further supports the conclusion that this dIN usAHP unmasked by blocking Ih is mediated by the recruitment of dynamic Na/K pumps.

**Figure 7:**
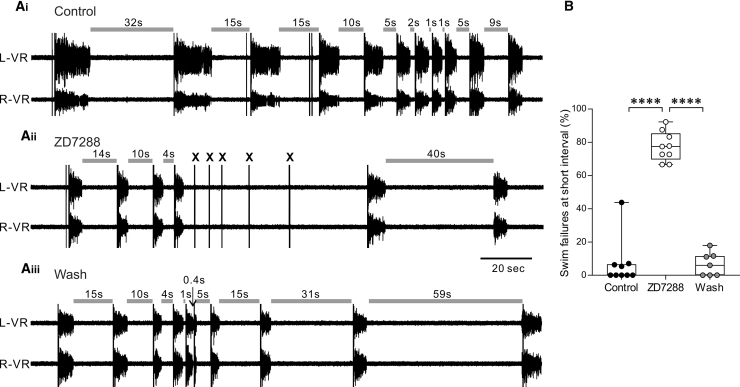
Block of Ih Current Disrupts the Relationship between Swim Interval and Episode Duration and Increases Swimming Failure at Short Intervals (A) Raw traces showing two simultaneously recorded ventral root traces on the left (L-VR) and right (R-VR) sides showing evoked swim episodes using variable inter-episode intervals. In control conditions (Ai), shortening the inter-episode interval reduces episode duration, but swimming can still be reliably evoked at very short intervals (≤2 s). In the presence of ZD7288, swimming initiation failed at short intervals (Aii; crosses, stimuli failing to evoke swimming), an effect which reversed upon drug washout (Aiii). (B) Pooled data illustrating that block of Ih significantly increases the swim failure rate at short inter-swim intervals (n = 6; median with 50% IQR displayed as box-and-whisker plot). ^∗∗∗∗^p < 0.0001.

A testable implication of the additional usAHP unmasked in dINs by block of Ih is that ZD7288 will impact the relationship between inter-swim interval and swim episode dynamics. For example, ZD7288 should compromise the animals’ ability to generate swimming at short inter-swim intervals, especially when the stimulus coincides with the trough of usAHPs in dINs. We therefore conducted a final series of experiments in which fictive swimming was evoked in a series of decreasing inter-swim intervals (cf. [22]), first in control saline and then in the presence of ZD7288 (Figure 7A; n = 9). After 20–30 min exposure to ZD7288, skin stimuli that reliably initiated swimming in control conditions regardless of inter-swim interval failed to elicit fictive swimming. The percentages of swim failures at intervals of up to around 5 s are pooled in Figure 7B (n = 6; control 2.7% ± 3.3%; ZD7288 75.3% ± 8%; wash 6.7% ± 7.1%); this effect of ZD7288 was reliable (n = 8/8) and reversible upon return to control saline (n = 6/8).

## Discussion

Ih currents are present in a wide range of networks, including those controlling rhythmic motor behaviors, and sculpt the intrinsic properties of neurons as well as being a target for neuromodulators. We have documented the presence of Ih in a specific class of tadpole swim CPG neurons—the excitatory dINs that drive the swimming rhythm—and demonstrate an important role for Ih in controlling dIN properties and swim network output.

### Selective Expression of Ih in dINs and Non-dINs

Ih was only present in a subset of non-dINs in the locomotor network of *Xenopus* tadpoles. Here, Ih was active only at very hyperpolarized, non-physiological membrane potentials and Ih block with ZD7288 had no effect on the RMP or input resistance of non-dINs. Ih current in this small subset of non-dINs could assist membrane potential repolarization and help to protect non-dINs from abnormal membrane hyperpolarization. Although Ih channels in non-dINs are unlikely to play a major role in locomotion at this early stage of larval development, their activation range may shift to more physiological membrane potentials later in *Xenopus* tadpole development, when Ih currents in spinal CPG neurons appear to be expressed more widely [39]. The Ih activation range in these neurons could also be shifted, for example, by changes in temperature or through neuromodulation [5]. Later in development, when spontaneous swimming episodes become more frequent [39], the usAHP and Ih in CPG neurons might dynamically interact with one another to set the regularity of spontaneous swimming.

In contrast, at the early larval stage of development described here, Ih currents were selectively and consistently present in dINs and occurred at physiologically relevant membrane potentials, being active at around −50 mV. Block of Ih with ZD7288 caused a large hyperpolarization of ∼10 mV, with an accompanying decrease in membrane conductance, demonstrating that Ih contributes to the characteristic depolarized RMP of the rhythm-generating neurons of the tadpole swim network [33]. The activation of Ih at rest in only a subset of CPG neurons (dINs) is not without precedent. For example, specific neurons of the leech heartbeat network, such as HNs and mechanosensory pressure neurons, show a prominent Ih at rest; but other neuron subtypes, such as Retzius neurons, display only a small sag potential at very hyperpolarized levels (>−70 mV) [40]. Again, the HNs in this network are the excitatory rhythm-generating interneurons, suggesting that Ih may play a common role in contributing to the rhythmicity of excitatory interneurons in phylogenetically diverse CPG networks.

### Role of Ih in Regulating *Xenopus* Locomotor Activity

We found that blocking Ih using ZD7288 (50 μM) had clear and significant effects on the swimming rhythm in *Xenopus* tadpoles. In particular, rhythmic swim episodes under ZD7288 were generally shorter and slower, with longer and more variable burst durations, compared to the typically stable and fast rhythm in control conditions. The lower concentration (10 μM) used for patch-clamp recordings also shortened swim episodes, although the cycles appeared largely unaffected (Figures 5A and 5B); this is most likely due to the shorter drug applications used for intracellular recordings and the lower concentration we applied to ensure effective washout of ZD7288. Previous studies have demonstrated similar slowing and/or disruptive effects of Ih blockade on rhythmic bursting. In the rhythmically active leech heartbeat network, Ih block causes tonic spiking in heartbeat interneurons, interspersed with periods of erratic, unstable bursting [7]. Conversely, enhancing Ih currents, either using the neuromodulator myomodulin or indirectly activating Ih by inhibiting sodium pump activity, increases the frequency of the rhythm [41]. Similar effects of Ih on rhythm frequency are observed in STG neurons of the lobster pyloric network [9, 42] and the swimming network of the sea angel, *Clione limacina* [10]. Thus, the presence of an Ih current appears to play a common role in rhythmic networks of stabilizing the rhythm and increasing cycle frequency, and the effects in the present study are consistent with this contribution of Ih.

We identified that the primary source of these effects of Ih block on the swim network is most likely the rhythm-generating dINs, although additional contributions aside from CPG network effects remain possible. Other CPG neurons (cINs, ascending interneurons, and motoneurons) are, however, unlikely to be directly involved. The precise mechanisms through which Ih block in dINs mediates the disruptive effects on swimming most likely occur on multiple levels. Critically, each cycle of swimming in *Xenopus* tadpoles is driven by single, synchronized spikes in the dIN population, which activates various CPG interneurons, including the inhibitory cIN population [32]. The cINs provide glycinergic, mid-cycle inhibition to the contralateral dIN population, which ensures unilateral excitation, but midcycle inhibitory postsynaptic potentials (IPSPs) from cINs also trigger PIR spikes in contralateral dINs to initiate the next swim cycle [32, 37]. The timing and duration of these IPSPs determines the frequency and stability of the locomotor rhythm [37]. Removal of Ih in dINs will therefore most likely have a number of consequences for this mechanism.

First, it is possible that Ih is activated and de-activated on a cycle-by-cycle basis in dINs and contributes dynamically to the amplitude or timing of PIR itself, as has been shown previously in other rhythmic networks [8, 43]. We showed that Ih is active within the peak membrane potential range reached by dINs during swimming in response to mid-cycle inhibition (∼−40 to −50 mV), suggesting that Ih not only contributes to the resting intrinsic properties in dINs but is also active during swimming in dINs. Indeed, we also found that following the hyperpolarization caused by Ih block, PIR spiking in dINs was largely abolished, supporting the idea that Ih contributes a key role toward dIN PIR firing during ongoing swimming. Thus, block of Ih may slow PIR firing in dINs, disturb the synchrony of dIN spikes, and disrupt rhythm generation, which is most likely responsible for the slower swim frequency, broader motor burst, and shorter swim episode, respectively. However, it is important to note that dINs did continue to spike during swimming even after Ih block, suggesting that other ionic mechanisms, such as de-inactivation of voltage-gated transient and persistent sodium channels, also play a contributing role. Despite firing rhythmically during swimming, all recorded dINs were depolarized to a more positive potential in response to tonic excitatory drive; such an enhanced voltage response may result from the increase in input resistance following Ih block. It is also important to note that removal of the hyperpolarization by washout of ZD7288, or depolarizing dINs to between −40 and −50 mV, can restore the ability to generate PIR spiking in response to hyperpolarization (Figures 5Ci and 5Cii).

Second, the large ZD7288-induced hyperpolarization of dINs will also affect their intrinsic excitability. For example, it will remove any resting Na^+^ channel inactivation [44] and thereby actually assist their spiking in response to subsequent depolarizing inputs. On the other hand, both the resetting of the membrane potential to a new more hyperpolarized level and the resulting usAHP following swimming could de-inactivate A-type K^+^ currents [23] that, in turn, would slow the rate of dIN depolarization and delay the onset of spiking. Any interruption to the precise timing of dIN spiking, which precedes firing in all other CPG neurons on each cycle [32], would interfere with the coordination of swimming, and this may account for the impairments reported here, including a deterioration in left-right coupling.

Finally, the removal of Ih will also most likely modify the responses of dINs to incoming synaptic inputs. For example, the presence of a resting Ih current reduces the amplitude and duration of incoming post-synaptic potentials, an effect which is critical in generating precisely timed spiking responses [6]. In CA1 pyramidal cells [45, 46, 47] and inner hair cell afferents [48], the duration of post-synaptic potentials is increased following Ih block, which has disruptive effects on spike integration by broadening the time window for synchronous inputs. In *Xenopus* tadpoles, such changes to the timing and duration of midcycle IPSPs will affect the precise timing of dIN firing and would be expected to slow the rhythm and lengthen burst durations. Unfortunately, it is not possible to accurately measure dIN IPSP durations during swimming in current clamp mode (see Figures 1C, 5Aii, and 5Bii), although further studies should test this idea using voltage-clamp recordings. Overall, it is likely that multiple mechanisms contribute to the effects of Ih block on the swim network, but our results suggest that dINs are the primary source of the effects.

### Interaction between Ih and Na/K Pump Current

Blocking Ih also unmasked a post-swim hyperpolarization in dINs, similar to a Na/K-pump-current-mediated usAHP in non-dINs. The fact that the pump current is revealed by ZD7288 in every dIN we recorded suggests that the mechanism proposed to be responsible for the usAHP (the presence of dynamic, activity-dependent α3-subunit containing Na/K pumps) [49] occurs in all dINs, in contrast to our previous assumption that it was entirely absent. A suprathreshold pulse train that mimics swimming induces a similar usAHP in only 66.7% of dINs tested, which is probably due to insufficient Na^+^ influx during induced action potentials, whereas during swimming, NMDA-receptor-mediated Na^+^ influx may play a major role in activating Na/K pumps. In leech heart interneurons, an interaction between Ih and Na/K pump current controls burst firing [24]; such a mechanism might also contribute toward dIN pacemaker-like firing during fictive swimming on a cycle-by-cycle basis. Furthermore, our data support the idea that Ih serves the homeostatic role of negating the hyperpolarizing influence that the dynamic pump current would otherwise have on dINs. This is potentially very important behaviorally because the activity-dependent pump current underlies a form of short-term motor memory that links future network output to past network performance [22]. Thus, if the network is reactivated after a short interval, the duration and intensity of the ensuing swim episode are reduced. However, it is important that the network always retains some residual rhythm-generating capability, so by compensating for the dynamic hyperpolarizing pump current, Ih confers dINs protection from the reduction of excitability the usAHP would otherwise impose.

Indeed, the unmasking of a usAHP in dINs by ZD7288 resulted in the failure to initiate fictive swimming at intervals below approximately 5 s, in stark contrast to the normal situation in which residual capacity to generate swimming activity is retained no matter how short the inter-swim interval (Figure 7) [22]. This supports the conclusion that the absence of a usAHP in dINs functions to protect the rhythm-generating neurons from activity-dependent hyperpolarization and preserves the ability to escape from potential threats, regardless of when the animal last swam. In conclusion, the selective expression of Ih in dINs ensures that the debilitating impact of a dIN usAHP on swim initiation is negated to protect the circuit from fatigue caused by repeated stimulation, as might be endured by repeated predation attempts.

In summary, excitatory dINs are the only members of the *Xenopus* larval swim network to express Ih within a normal physiological range, and Ih is active in these neurons at rest. During larval locomotor rhythm generation, Ih appears to play three crucial roles: (1) contributing to dIN passive properties; (2) ensuring precise dIN rebound firing; and (3) counteracting the dynamic Na/K pump current mediating the usAHP.

## STAR★Methods

### Key Resources Table

**Table:** 

REAGENT or RESOURCE	SOURCE	IDENTIFIER
**Chemicals, Peptides, and Recombinant Proteins**		

ZD7288	abcam	ab120102
α-bungarotoxin	Invitrogen	B-1601
human chorionic gonadotropin	Sigma -Aldrich	CG10
neurobiotin	Vector lab	SP-1120

**Experimental Models: Organisms/Strains**		

*Xenopus laevis* wild type	Animal colonies; University of St Andrews; University of Edinburgh	N/A

**Software and Algorithms**		

DataView	Dr W. Heitler; University of St Andrews	N/A

### Contact for Reagent and Resource Sharing

The Key Resources Table lists resources used here. Further information and requests for resources and reagents should be directed to and will be fulfilled by the Lead Contact, Hong-Yan Zhang (hongyan.zhang@ed.ac.uk).

### Experimental Model and Subject Details

All experiments conformed to UK Home Office regulations and were approved by the Animal Welfare Ethics Committee (AWEC) of the University of St Andrews and University of Edinburgh. All experiments were performed on newly hatched pre-feeding *Xenopus laevis* tadpoles at developmental stage 42 [50]. Tadpoles were reared from fertilized ova obtained following breeding of adults selected from in-house colonies. Mating was induced by injections of human chorionic gonadotropin (HCG, 1000 U/mL, Sigma, UK) into the dorsal lymph sac of breeding pairs of adult frogs.

### Method Details

#### Electrophysiology

*Xenopus* tadpoles were immobilized by placing in 12.5 μM α-bungarotoxin saline for approximately 30 min, and then mounted on a rotatable Sylgard platform in a bath of saline (in mM: 115 NaCl, 2.5 KCl, 2 CaCl_2_, 2.4 NaHCO_3_, 1 MgCl_2_, 10 HEPES, adjusted with 4 M NaOH to pH 7.4). Both sides of the trunk skin overlying the myotomal muscles were removed using a finely etched needle and forceps. The extracellular ventral root activities (fictive swimming) were recorded using one or two suction electrodes placed on the cleft between two trunk muscle blocks. The dorsal parts of approximately 7 rostral myotomes were freed from the spinal cord and the roof of the hindbrain and spinal cord was opened to the neurocoel to improve drug access and provide access for patch clamp electrodes.

Exposed neuronal somata were patch clamped using borosilicate glass pipettes (Harvard Apparatus Ltd) pulled on a Sutter P97 pipette puller. Patch pipettes were filled with 0.1% neurobiotin (Vector lab) in the intracellular solution (in mM: 100 K-gluconate, 2 MgCl_2_, 10 EGTA, 10 HEPES, 3 Na_2_ATP, 0.5 NaGTP adjusted to pH 7.3 with KOH) and had resistances of ∼10 MΩ. Changes in membrane potential were recorded in current clamp mode using an Axoclamp 2B or MultiClamp 700B amplifier. Simultaneous extracellular recordings of fictive swimming were made with suction electrodes from ventral roots at intermyotomal clefts, and signals were amplified using differential AC amplifiers (A-M Systems Model 1700). Simultaneous intracellular and extracellular signals were digitized using a CED Power 1401, and displayed and stored on a PC computer using Spike2 or Signal software. Fictive swimming was initiated by stimulating through a glass suction electrode placed on the tail skin, which delivered a 1 ms current pulse via a DS2A isolated stimulator (Digitimer). A rest time of 2 min was allowed between evoked episodes of swimming to ensure each episode was not influenced by preceding activity [22, 23].

Patch-clamp recordings typically had 20-30 min control period and then continued for about 15-20 min in the presence of 10 μM ZD7288, which was followed by a washout period for 30-60 min. When ouabain or zero K^+^ saline was applied, another 10-20 min treatment period was added before the washout (with ZD7288 still present). The ventral root recordings consisted of at least 30 min of control period with regular stimulation of swimming at 2 min intervals. The treatment period (50 μM ZD7288) lasted 30-45 min in order for the drug to fully penetrate the tissue as only trunk skin was removed from the otherwise intact tadpoles used in these experiments. The washout period lasted approximately 1 hr, therefore allowing a much better, albeit not full, reversal of effects. All drugs were bath-applied.

#### Neuron identification

Following each patch-clamp recording, animals were fixed in 2% glutaraldehyde in 0.1 M phosphate buffer, pH 7.2, overnight in a refrigerator (∼4°C). Animals were first rinsed with 0.1 M PBS (120 mM NaCl in 0.1 M phosphate buffer, pH 7.2), and washed in two changes of 1% Triton X-100 in PBS for 15 min with agitation. Next, animals were incubated in a 1:300 dilution of extravidin peroxidase conjugate in PBS containing 0.5% Triton X-100 for 2–3 hr with agitation, and washed again in at least four changes of PBS. Animals were then immersed in 0.08% diaminobenzidine in 0.1 M PBS (DAB solution) for 5 min, moved to a DAB solution with 0.075% hydrogen peroxide for 1-2 min, and then washed in running tap water. Finally, animals were dehydrated in 100% alcohol, cleared in methyl benzoate and xylene, and mounted whole, between two coverslips using Depex. A small proportion of recorded neurons were damaged while withdrawing the patch electrode and/or during the staining procedure, and could not be visualized. Neuronal cell bodies and axon processes were observed under a x40 objective to identify CPG neuron types. All reagents were obtained from Sigma or Tocris Bioscience.

### Quantification and Statistical Analysis

Electrophysiological data were first analyzed using DataView software (v10.3.0, courtesy of Dr. W. J. Heitler) and all raw data were imported into Excel spreadsheets and analyzed. Statistical analyses were conducted using PASW statistics 21 or Prism 6. For swim episode duration analysis, we calculated a mean of 3 consecutive evoked episodes in each condition. For intra-episode swim parameters (cycle frequency, burst duration) a mean of the first 20 cycles of swimming across an episode in each condition was calculated. Swim burst durations were identified by applying a threshold to the rectified and integrated trace. The start and the end of a burst was defined as the onset and offset of the threshold crossing. The tonic depolarization levels were measures at the 10th action potential of each swimming episode. For all experiments, values are stated as mean ± SD and displayed as box-and-whisker plots with individual experiments plotted as data points. Unless otherwise stated conditions were compared using either paired t tests or repeated-measures ANOVAs followed by Bonferonni-corrected post hoc comparison. All tests were 2-tailed, and n numbers are reported in both results and figure legends. For ventral root recordings, each n is data from one animal. For patch recordings, each n comes from a single neuron from one animal.

### Data and Software Availability

Further information and requests for datasets and analysis software should be directed to Hong-Yan Zhang, (hongyan.zhang@ed.ac.uk).

## Supplemental Information

Document S1. Figures S1–S3

Document S2. Article plus Supplemental Information

## References

[1] Craven K.B., Zagotta W.N. (2006). CNG and HCN channels: two peas, one pod. Annu. Rev. Physiol..

[2] Noma A., Irisawa H. (1976). Membrane currents in the rabbit sinoatrial node cell as studied by the double microelectrode method. Pflugers Arch..

[3] Moosmang S., Stieber J., Zong X., Biel M., Hofmann F., Ludwig A. (2001). Cellular expression and functional characterization of four hyperpolarization-activated pacemaker channels in cardiac and neuronal tissues. Eur. J. Biochem..

[4] Pape H.-C. (1996). Queer current and pacemaker: the hyperpolarization-activated cation current in neurons. Annu. Rev. Physiol..

[5] Robinson R.B., Siegelbaum S.A. (2003). Hyperpolarization-activated cation currents: from molecules to physiological function. Annu. Rev. Physiol..

[6] Biel M., Wahl-Schott C., Michalakis S., Zong X. (2009). Hyperpolarization-activated cation channels: from genes to function. Physiol. Rev..

[7] Angstadt J.D., Calabrese R.L. (1989). A hyperpolarization-activated inward current in heart interneurons of the medicinal leech. J. Neurosci..

[8] Harris-Warrick R.M., Coniglio L.M., Levini R.M., Gueron S., Guckenheimer J. (1995). Dopamine modulation of two subthreshold currents produces phase shifts in activity of an identified motoneuron. J. Neurophysiol..

[9] Peck J.H., Gaier E., Stevens E., Repicky S., Harris-Warrick R.M. (2006). Amine modulation of *I*_h_ in a small neural network. J. Neurophysiol..

[10] Pirtle T.J., Satterlie R.A. (2007). The role of postinhibitory rebound in the locomotor central-pattern generator of *Clione limacina*. Integr. Comp. Biol..

[11] Thoby-Brisson M., Telgkamp P., Ramirez J.M. (2000). The role of the hyperpolarization-activated current in modulating rhythmic activity in the isolated respiratory network of mice. J. Neurosci..

[12] McCormick D.A., Pape H.C. (1990). Properties of a hyperpolarization-activated cation current and its role in rhythmic oscillation in thalamic relay neurones. J. Physiol..

[13] Maccaferri G., McBain C.J. (1996). The hyperpolarization-activated current (*I*_h_) and its contribution to pacemaker activity in rat CA1 hippocampal stratum oriens-alveus interneurones. J. Physiol..

[14] Harris-Warrick R.M. (2011). Neuromodulation and flexibility in central pattern generator networks. Curr. Opin. Neurobiol..

[15] Smith M., Perrier J.-F. (2006). Intrinsic properties shape the firing pattern of ventral horn interneurons from the spinal cord of the adult turtle. J. Neurophysiol..

[16] Butt S.J.B., Harris-Warrick R.M., Kiehn O. (2002). Firing properties of identified interneuron populations in the mammalian hindlimb central pattern generator. J. Neurosci..

[17] Kiehn O., Kjaerulff O., Tresch M.C., Harris-Warrick R.M. (2000). Contributions of intrinsic motor neuron properties to the production of rhythmic motor output in the mammalian spinal cord. Brain Res. Bull..

[18] Takahashi T. (1990). Inward rectification in neonatal rat spinal motoneurones. J. Physiol..

[19] Moult P.R., Cottrell G.A., Li W.C. (2013). Fast silencing reveals a lost role for reciprocal inhibition in locomotion. Neuron.

[20] Hull M.J., Soffe S.R., Willshaw D.J., Roberts A. (2016). Modelling feedback excitation, pacemaker properties and sensory switching of electrically coupled brainstem neurons controlling rhythmic activity. PLoS Comput. Biol..

[21] Svensson E., Jeffreys H., Li W.-C. (2017). The modulation of two motor behaviors by persistent sodium currents in *Xenopus laevis* tadpoles. J. Neurophysiol..

[22] Zhang H.-Y., Sillar K.T. (2012). Short-term memory of motor network performance via activity-dependent potentiation of Na^+^/K^+^ pump function. Curr. Biol..

[23] Zhang H.-Y., Picton L., Li W.C., Sillar K.T. (2015). Mechanisms underlying the activity-dependent regulation of locomotor network performance by the Na^+^ pump. Sci. Rep..

[24] Kueh D., Barnett W.H., Cymbalyuk G.S., Calabrese R.L. (2016). Na^+^/K^+^ pump interacts with the *h*-current to control bursting activity in central pattern generator neurons of leeches. eLife.

[25] Kim J.H., von Gersdorff H. (2012). Suppression of spikes during posttetanic hyperpolarization in auditory neurons: the role of temperature, *I*_h_ currents, and the Na^+^-K^+^-ATPase pump. J. Neurophysiol..

[26] Gulledge A.T., Dasari S., Onoue K., Stephens E.K., Hasse J.M., Avesar D. (2013). A sodium-pump-mediated afterhyperpolarization in pyramidal neurons. J. Neurosci..

[27] Robert A., Jirounek P. (1998). Axonal and glial currents activated during the post-tetanic hyperpolarization in non-myelinated nerve. Pflugers Arch..

[28] Baginskas A., Palani D., Chiu K., Raastad M. (2009). The H-current secures action potential transmission at high frequencies in rat cerebellar parallel fibers. Eur. J. Neurosci..

[29] Rozzo A., Ballerini L., Abbate G., Nistri A. (2002). Experimental and modeling studies of novel bursts induced by blocking Na^+^ pump and synaptic inhibition in the rat spinal cord. J. Neurophysiol..

[30] Kang Y., Notomi T., Saito M., Zhang W., Shigemoto R. (2004). Bidirectional interactions between h-channels and Na^+^-K^+^ pumps in mesencephalic trigeminal neurons. J. Neurosci..

[31] Kiehn O. (2016). Decoding the organization of spinal circuits that control locomotion. Nat. Rev. Neurosci..

[32] Roberts A., Li W.-C., Soffe S.R. (2010). How neurons generate behavior in a hatchling amphibian tadpole: an outline. Front. Behav. Neurosci..

[33] Sautois B., Soffe S.R., Li W.C., Roberts A. (2007). Role of type-specific neuron properties in a spinal cord motor network. J. Comput. Neurosci..

[34] Sillar K.T., Wedderburn J.F.S., Simmers A.J. (1991). The development of swimming rhythmicity in post-embryonic *Xenopus laevis*. Proc. Biol. Sci..

[35] Zhang H.-Y., Issberner J., Sillar K.T. (2011). Development of a spinal locomotor rheostat. Proc. Natl. Acad. Sci. USA.

[36] Darbon P., Yvon C., Legrand J.C., Streit J. (2004). INaP underlies intrinsic spiking and rhythm generation in networks of cultured rat spinal cord neurons. Eur. J. Neurosci..

[37] Li W.-C., Moult P.R. (2012). The control of locomotor frequency by excitation and inhibition. J. Neurosci..

[38] Li W.-C., Soffe S.R., Wolf E., Roberts A. (2006). Persistent responses to brief stimuli: feedback excitation among brainstem neurons. J. Neurosci..

[39] Currie S.P., Sillar K.T. (2018). Developmental changes in spinal neuronal properties, motor network configuration, and neuromodulation at free-swimming stages of *Xenopus* tadpoles. J. Neurophysiol..

[40] Gerard E., Hochstrate P., Dierkes P.-W., Coulon P. (2012). Functional properties and cell type specific distribution of *I*_h_ channels in leech neurons. J. Exp. Biol..

[41] Tobin A.-E., Calabrese R.L. (2005). Myomodulin increases *I*_h_ and inhibits the Na/K pump to modulate bursting in leech heart interneurons. J. Neurophysiol..

[42] Zhang Y., Oliva R., Gisselmann G., Hatt H., Guckenheimer J., Harris-Warrick R.M. (2003). Overexpression of a hyperpolarization-activated cation current (*I*_h_) channel gene modifies the firing activity of identified motor neurons in a small neural network. J. Neurosci..

[43] Pirtle T.J., Willingham K., Satterlie R.A. (2010). A hyperpolarization-activated inward current alters swim frequency of the pteropod mollusk *Clione limacina*. Comp. Biochem. Physiol. A Mol. Integr. Physiol..

[44] Kuo C.C., Bean B.P. (1994). Na^+^ channels must deactivate to recover from inactivation. Neuron.

[45] Hardie J.B., Pearce R.A. (2006). Active and passive membrane properties and intrinsic kinetics shape synaptic inhibition in hippocampal CA1 pyramidal neurons. J. Neurosci..

[46] Magee J.C. (1998). Dendritic hyperpolarization-activated currents modify the integrative properties of hippocampal CA1 pyramidal neurons. J. Neurosci..

[47] Pavlov I., Scimemi A., Savtchenko L., Kullmann D.M., Walker M.C. (2011). I_h_-mediated depolarization enhances the temporal precision of neuronal integration. Nat. Commun..

[48] Yi E., Roux I., Glowatzki E. (2010). Dendritic HCN channels shape excitatory postsynaptic potentials at the inner hair cell afferent synapse in the mammalian cochlea. J. Neurophysiol..

[49] Picton L.D., Nascimento F., Broadhead M.J., Sillar K.T., Miles G.B. (2017). Sodium pumps mediate activity-dependent changes in mammalian motor networks. J. Neurosci..

[50] Nieuwkoop P.D., Faber J. (1994). Normal Table of *Xenopus laevis* (Daudin).

