# Supplementary material for: Control of Xenopus Tadpole Locomotion via Selective Expression of Ih in Excitatory Interneurons
Source: Curr Biol. 2018 Dec 17;28(24):3911–3923.e2. doi: 10.1016/j.cub.2018.10.048 (PMC6303192; doi:10.1016/j.cub.2018.10.048)
Supplement: Document S1. Figures S1–S3 [file mmc1.pdf]

Current Biology, Volume *28*

Supplemental Information

Control of *Xenopus* Tadpole Locomotion via Selective Expression of Ih in Excitatory Interneurons

Laurence D. Picton, Keith T. Sillar, and Hong-Yan Zhang

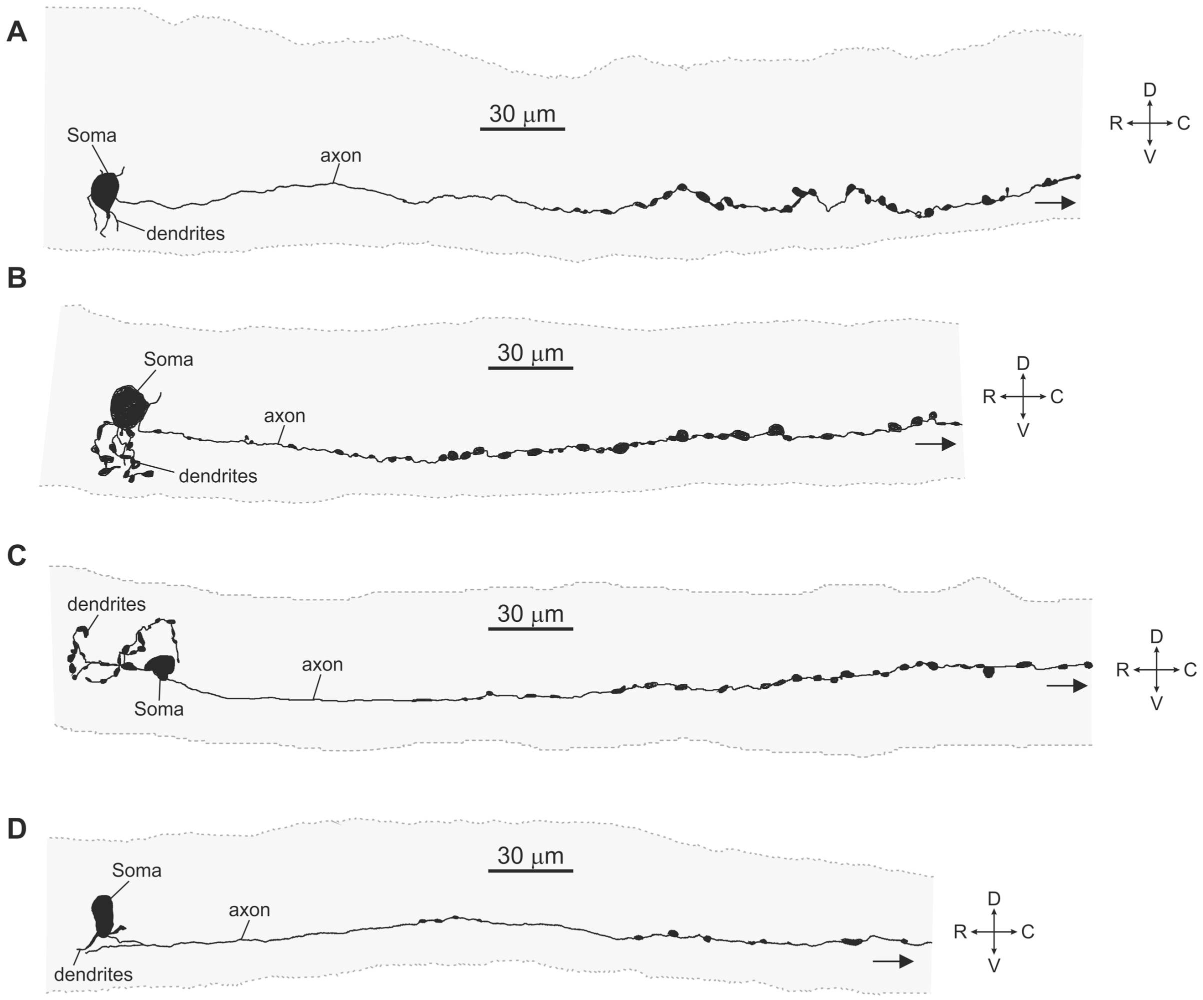

Figure S1. The Anatomy of larval dINs. Related to Figure 1.

(A-D) Four additional examples of larval dINs at stage 42. All successfully stained dINs at this stage displayed a long, thin descending axon that runs ipsilateral to a multipolar soma with short dendrites. Only a very short ascending axon was ever observed (e.g. in D).

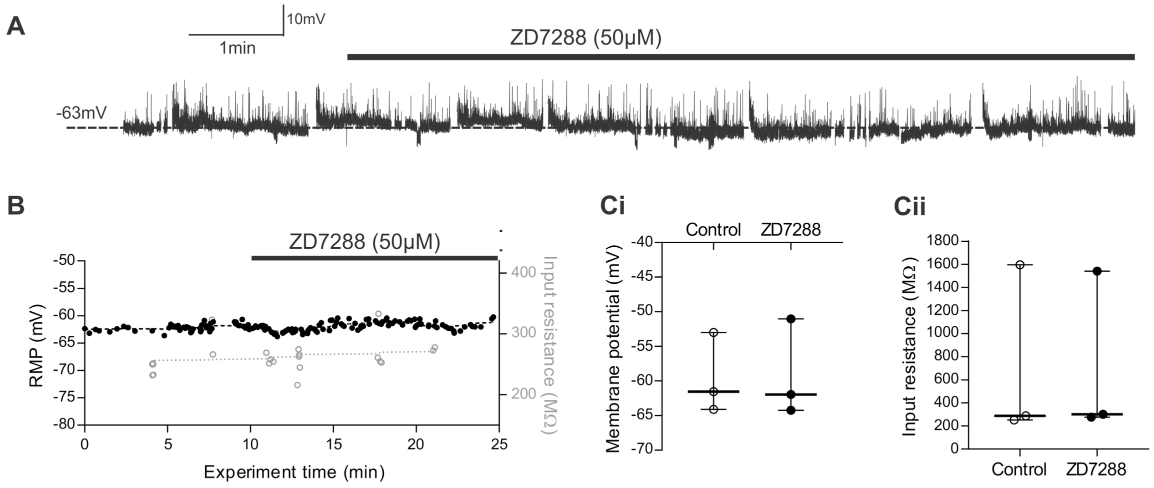

Figure S2. Block of Ih in Non-dINs does not Affect Their Intrinsic Properties. Related to Figure 2 and 3.

(A) Slow timebase recording of a non-dIN neuron showing the lack of effect of ZD7288 (50 µM) on membrane potential. Note gaps in this continuous raw recording due to post hoc removal of applied current protocols.

(B) The resting membrane potential (RMP) and input resistance (IR) of the cell shown in (A) plotted against experiment time.

(C) There was no overall effect of ZD7288 on RMP (Ci: p=0.93, n=3) or input resistance (Cii, p=0.99, n=3) in non-dIN neurons displaying a sag potential. Data are expressed as median with 50% IQR and displayed as box-and-whisker plots with individual data points.

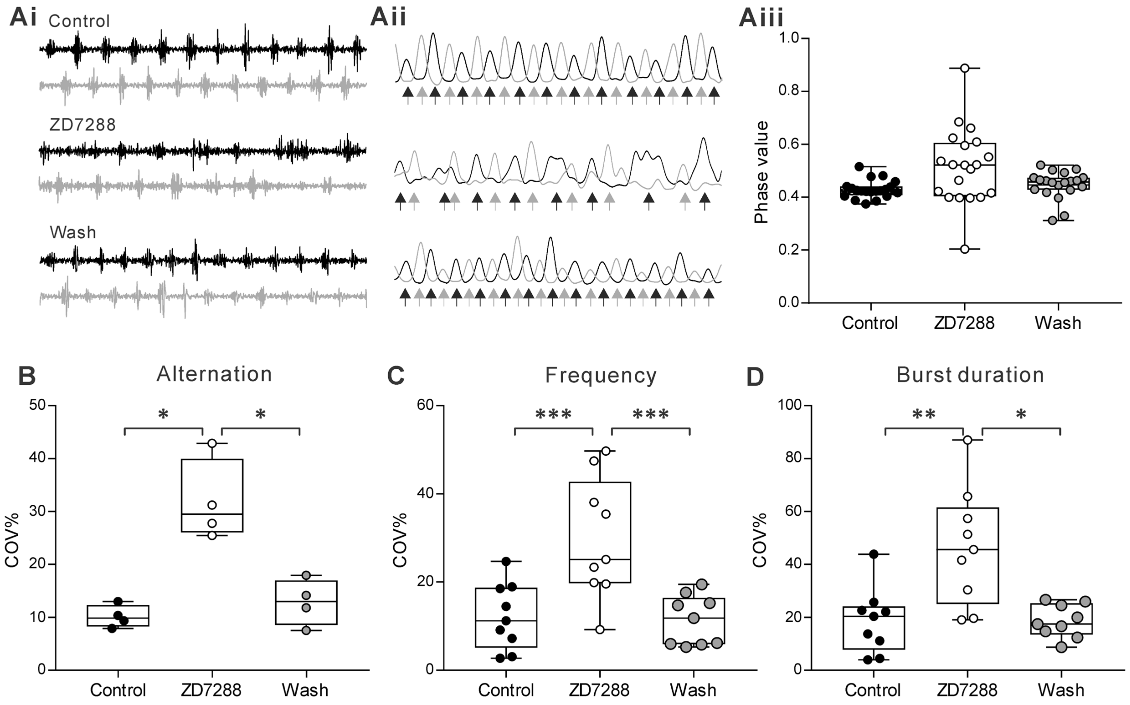

Figure S3. ZD7288 Increased the Variation of Several Parameters of *Xenopus* Swimming Activity. Related to Figure 4.

(Ai) Two simultaneously recorded raw ventral root traces on the left and rights sides showing evoked swim episodes in control, in the presence of the Ih current blocker ZD7288 (50 μM) and after washout.

(Aii) Rectified/integrated traces from raw traces in (Ai) clearly showing a disturbance in left-right alternation.

(Aiii) An example of the phase values in a single experiment. ZD7288 (50 μM) did not completely disrupt the coordination between the left and right sides, but the variation of phase values increased. Data are expressed as median with 50% IQR displayed as box-and-whisker plots with individual data points.

(B-D) The coefficient of variation (COV%) was significantly increased by ZD7288 for the left/right cycle phase (B; P=0.03), the swimming frequency (C; P=0.0005) and the burst durations (D; P=0.0012) (n=9). *, P<0.05; **, P<0.01; ***, P<0.001. Data are expressed as median with 50% IQR and displayed as box-and-whisker plots with individual data points.
